# Phylogenetic Analysis of Core Melanin Synthesis Genes Provides Novel Insights Into the Molecular Basis of Albinism in Fish

**DOI:** 10.3389/fgene.2021.707228

**Published:** 2021-08-04

**Authors:** Chao Bian, Ruihan Li, Zhengyong Wen, Wei Ge, Qiong Shi

**Affiliations:** ^1^Faculty of Health Sciences, Centre of Reproduction, Development and Aging, University of Macau, Taipa, China; ^2^Shenzhen Key Lab of Marine Genomics, Guangdong Provincial Key Lab of Molecular Breeding in Marine Economic Animals, Beijing Genomics Institute, BGI Academy of Marine Sciences, BGI Marine, Shenzhen, China; ^3^College of Life Sciences, University of Chinese Academy of Sciences, Beijing, China

**Keywords:** melanin synthesis pathway, core genes for melanin synthesis, albinism phenotype, nonsense mutation, phylogenetic analysis

## Abstract

Melanin is the most prevalent pigment in animals. Its synthesis involves a series of functional genes. Particularly, teleosts have more copies of these genes related to the melanin synthesis than tetrapods. Despite the increasing number of available vertebrate genomes, a few systematically genomic studies were reported to identify and compare these core genes for the melanin synthesis. Here, we performed a comparative genomic analysis on several core genes, including tyrosinase genes (*tyr*, *tyrp1*, and *tyrp2*), premelanosome protein (*pmel*), microphthalmia-associated transcription factor (*mitf*), and solute carrier family 24 member 5 (*slc24a5*), based on 90 representative vertebrate genomes. Gene number and mutation identification suggest that loss-of-function mutations in these core genes may interact to generate an albinism phenotype. We found nonsense mutations in *tyrp1a* and *pmelb* of an albino golden-line barbel fish, in *pmelb* of an albino deep-sea snailfish (*Pseudoliparis swirei*), in *slc24a5* of cave-restricted Mexican tetra (*Astyanax mexicanus*, cavefish population), and in *mitf* of a transparent icefish (*Protosalanx hyalocranius*). Convergent evolution may explain this phenomenon since nonsense mutations in these core genes for melanin synthesis have been identified across diverse albino fishes. These newly identified nonsense mutations and gene loss will provide molecular guidance for ornamental fish breeding, further enhancing our in-depth understanding of human skin coloration.

## Introduction

Pigment patterns and coloration of skin, feathers, hair and scales are among the most variable phenotypes in various vertebrates (Protas and Patel, 2008). Diverse coloration phenotypes present some substantial functions in mate selection, crypsis, aposematism of predators, and species recognition (Protas and Patel, 2008). Melanin is the most prevalent pigment in animals with the primary function of shielding against UV irradiation from sunlight. It is synthesized in pigment cells or chromatophores that are derived from neural crests (Singh and Nüsslein-Volhard, 2015). Mammals and birds exhibit only one category of pigment cells, also named melanocytes including black and brown types. Reptiles and amphibians possess xanthophores and iridophores. In fishes, there are seven different types of pigment cells, including melanophores, xanthophores, iridophores, erythrophores, leucophores, cyanophores and erythro-iridophores (Nordlund, 1992; Singh and Nüsslein-Volhard, 2015).

**FIGURE 1:**
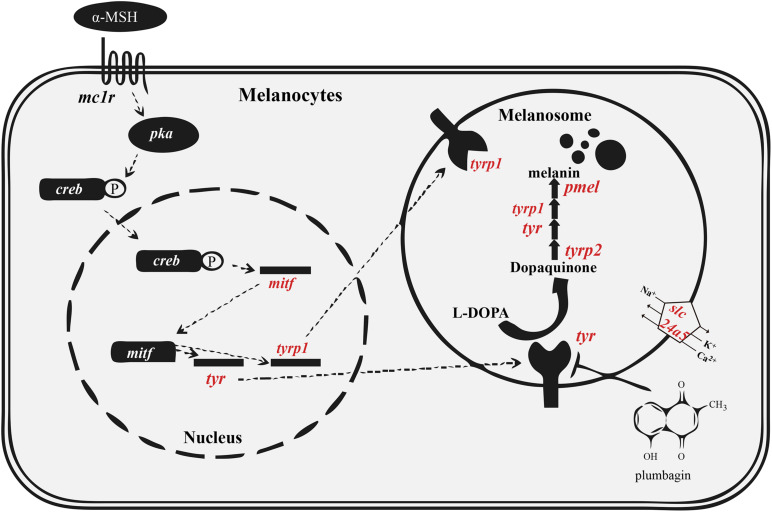
Melanin synthesis pathway in vertebrates. The tyrosinase family (*tyr*, *tyrp1* and *tyrp2*) and *pmel* are essential for melanin synthesis. The melanosomal transporter (*slc24a5*) and regulatory factor *(mitf)* are also important for proper melanin synthesis. Red indicates the genes that we analyzed in detail. This figure was modified from a previous report (Oh et al., 2017). Abbreviations: α-MSH, alpha-melanocyte stimulating hormone; *creb*, cAMP response element binding protein; *mc1r*, melanocortin receptor; *pka*, protein kinase A. See other abbreviations in corresponding text.

The black pigment, also named melanin, is generated from tyrosine in the melanosome (del Marmol and Beermann, 1996). Two types of melanin are present in mammals and birds, including black eumelanin and lighter pheomelanin. In various fishes, however, only eumelanin is found (Burton, 2011). The detailed melanin synthesis pathway is summarized in Figure 1. Disruption of the melanogenesis process causes decreased pigmentation, which may lead to complete absence of melanin (Braasch et al., 2007).

The genetic basis of pigment cell development and differentiation is largely conserved between tetrapods and fishes. It is well documented that a big proportion of genes in the melanin synthesis pathway have been duplicated and differentially retained in teleosts (Braasch et al., 2007, 2009a), possibly due to the third round of whole genome duplication (also known as the teleost-specific whole genome duplication, TWGD) approximately 250–350 million years ago (Mya) (Jaillon et al., 2004). Therefore, teleosts usually possess more melanin synthesis genes than tetrapods. Melanin biosynthesis in vertebrates depends on three members of the tyrosinase family, including tyrosinase (*tyr*), and tyrosinase-related protein 1 and 2 (*tyrp1* and *tyrp2*). The *tyrp1* gene might play an additional role in survival and proliferation of melanocytes. The expression of *pmel* (premelanosome protein) and *tyrp1* genes is often regulated by *mitf* (microphthalmia-associated transcription factor a; see Figure 1; Braasch et al., 2009a; Bian et al., 2020).

The albinism phenotype (loss of melanin) is a fascinating trait of ornamental fishes, such as glass catfish, white cichlid fish and albino northern snakehead fish. The *casper* zebrafish (named after its ghost like appearance; White et al., 2008) and *casper* stickleback (Hart and Miller, 2017), displaying reduced pigmentation of melanosomes, can also be useful models for tumor engraftment and *in vivo* stem cell analyses with high sensitivity and resolution. Therefore, it is important to understand the detailed molecular mechanism and core genes involved in melanin biosynthesis, which can improve the efficiency of color breeding in ornamental fishes. This can also help to generate a wide variety of species to act as excellent research models. With an increasing number of available vertebrate genome sequences, we can analyze and identify core genes for melanin synthesis in various vertebrates. In this study, we analyzed genes involved in the melanin synthesis pathway in 90 representative vertebrates, including mammals, birds, reptiles, amphibians, Actinopterygii, and Chondrichthyes. For our better understanding of the melanin synthesis pathway in vertebrates, we explored several core genes in this pathway, such as *tyr*, *tyrp1*, *tyrp2*, *pmel*, *mitf*, and *solute carrier family 24 member 5* (*slc24a5*), in various vertebrates.

## Materials and Methods

### Genome Data Collection and Gene Identification

In total, 90 representative vertebrate genome assemblies were selected. They were downloaded from the National Center for Biotechnology Information (NCBI; Supplementary Table 1). The scaffold N50 data of these genome assemblies was also shown in Supplementary Table 1. Each genome sequence was prepared for construction in a standard BLAST database for subsequent BLAST analyses. The published protein sequences of *tyra*, *tyrb*, *tyrp1*, *tyrp2*, *pmela*, *pmelb*, *mitfa*, *mitfb*, and *slc24a5* from reference genomes (Japanese medaka *Oryzias latipes*, human *Homo sapiens*, chicken *Gallus gallus*, African clawed frog *Xenopus laevis*, and green Anoli lizard *Anolis carolinensiswere*) were downloaded from the public Ensembl database (Supplementary Table 2).

In detail, the protein sequences of medaka, human, chicken, frog, and green lizard were used for aligning the assemblies of Actinopterygii, mammals, birds, amphibians, and reptile species by using tBLASTn (version 2.2.28, NCBI, Bethesda, MD, United States) (Mount, 2007) with an E-value of 10^–5^. These alignments were further filtered and processed by using a Perl script to generate best-hit genomic regions containing putative target genes of each genome assembly with over 50% identity and aligned ratio. GeneWise v2.2.0 (Birney et al., 2004) was employed to predict target gene structures in the best-hit alignments from the 90 representative vertebrate genomes. Most importantly, if this pipeline identified a gene loss in any examined species, we then manually checked the tBLASTn alignments in this species to make sure that the data were indeed true. The gene was examined again in the final gene list from the genome annotation, if it has completed gene structure but its alignment identity and aligned ratio were between 45 and 50%. This lower identity and align ratio could be caused by rapid evolutionary rate of this gene in some species. Meanwhile, we also checked for synteny in genes form up and downstream of these genes as an additional indicator of orthology. Detailed copy numbers of above indicated genes in 90 vertebrate genomes and 9 representative genomes are provided in Tables 1, 2 and Supplementary Tables 3, 4.

**TABLE 1:** Copy numbers of representative genes for melanin synthesis in 90 representative vertebrate genomes.

Class	Species number	*tyr*		*tyrp*		*tyrp2*	*pmel*		*mitf*		*slc24a5*
		*tyra*	*tyrb*	*tyrp1a*	*tyrp1b*		*pmela*	*pmelb*	*mitfa*	*mitfb*	
Actinopterygii	36	37	29	37	36	38 (2)	40 (1)	37 (4)	39 (1)	41	38 (1)
Birds	25	25		25		23 (2)	5		25		25
Amphibians	3	3		5		4	4		4		4
Mammals	13	11		12		12 (1)	13		13		13
Reptiles	12	9		12		13	8		12		12
Chondrichthyes	1	1		1		1	1		1		1

*The data in brackets are the predicted copy numbers of pseudogenes.*

**TABLE 2:**
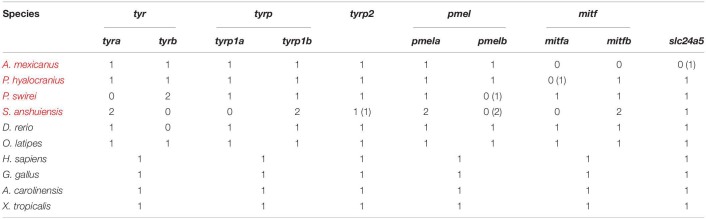
Copy numbers of representative genes for melanin synthesis in 9 representative vertebrate species.

*Species with melanin loss are marked in red. Predicted copy numbers of pseudogenes are provided in brackets.*

### Phylogenetic Analysis

Phylogenetic analysis was performed on the protein sequences. Multiple sequence alignments of amino acid sequences were conducted across various species using the Muscle software (Kumar et al., 2016). The trimAl software (Capella-Gutierrez et al., 2009) with default parameters was employed for alignment trimming. Phylogenetic trees were constructed by PhyML (version 3.1) (Guindon et al., 2010) with the amino acid substitution model of JTT + I + G and the maximum likelihood (ML) method. The protein sequences of ghost shark (*Callorhynchus milii*) were used as the outgroups. A total of 1,000 bootstrap replicates were applied for evaluation of their branch supports. The trees were displayed by FigTree.^1^

### Pseudogene Identification and Protein Sequence Alignment

We focused on identification of the core genes for melanin synthesis in several representative species with a loss-of-melanin phenotype. These vertebrates included *Astyanax mexicanus*, *Sinocyclocheilus anshuiensis, Protosalanx hyalocranius*, *Pseudoliparis swirei*, and *Danio rerio* (*nacre/casper* mutants). The corresponding nucleotide sequences of three-spined stickleback (*Gasterosteus aculeatus*), *D. rerio* (*Tu* strain), *Oryzias latipes, Anolis carolinensis, Homo sapiens*, and *Gallus gallus* were used as references for comparison. Multiple sequence alignments of the above-mentioned species were produced with the Muscle software in MEGA package (Kumar et al., 2016). Codon-based alignments were utilized to examine irregular shifts in the open reading frame for identification of possible pseudogenes, which were characterized by codon frameshifts or premature stop codon(s).

## Results

We screened 90 vertebrate genomes for *tyr*, *tyrp1*, *tyrp2*, *pmel*, *mitf*, and *slc24a5* genes, representing 36 Actinopterygii species, 13 mammals, 25 birds, 12 reptiles, three amphibians, and one chondrichthyan fish. Copy numbers of each gene are summarized in Table 1.

### Tyrosinase Gene Family

Melanin synthesis in vertebrates involves several important tyrosinase family members, including *tyr*, *tyrp1*, and *tyrp2* (Oetting and Setaluri, 2007). The *tyr* and *tyrp1* genes have been doubled and subsequently preserved in the teleost ancestor after the TWGD. The protein product of *tyr* catalyzes the first two rate-limiting steps of melanin synthesis by converting tyrosine to DOPA, and then catalyzes dopaquinone to melanin (Oetting and Setaluri, 2007). Mutations in the *tyr* gene of the *sandy* zebrafish strain caused melanin loss (Kelsh et al., 1996). Similarly, mutations in *tyr* lead to oculocutaneous albinism type 1 (OCA1) in humans (Dessinioti et al., 2009). The protein product of *tyrp2* catalyzes dopachrome to DHI-2-carboxylic acid (DHICA), while the *tyrp1* could participate not only in stabilizing the *tyr* in melanosome membranes (Kobayashi and Hearing, 2007; Krauss et al., 2014), but also in the formation of indole-5,6-quinone carboxylic acid from DHICA. A mutation in the coding region of *tyrp1a* caused melanophore death in zebrafish (Krauss et al., 2014). The double knock-down experiment of *tyrp1a* and *tyrp1b* in zebrafish led to hypo-pigmented melanophores and brown pigment (Braasch et al., 2009b). The oculocutaneous albinism type 3 (OCA3) of human, also known as Rufous albinism, is caused by mutations (Arg93Cys) in *tyrp1* (Dessinioti et al., 2009). The mutations of *tyrp1* gene also generally caused chocolate or brown coat color in many mammals and birds, like mice (Zdarsky et al., 1990), dog (Hrckova Turnova et al., 2017), chicken (Li et al., 2019), and Japanese quail (Nadeau et al., 2007).

We found that most of the examined teleost fishes possessed two copies of *tyr* (*tyra and tyrb*) and *tyrp1* (*tyrp1a* and *tyrp1b*), but only one copy of *tyrp2*. However, in all examined actinopterygian genomes, only the cave-restricted *S. anshuiensis* lost the *tyrp1a* gene; however, nine fish species have lost the *tyrb* gene (see more details in Supplementary Table 3). Both pufferfishes (fugu and Tetraodon) have lost the *tyrp1b* gene, which is consistent with a previous study (Braasch et al., 2007). The three Chinese golden-line barbel fishes (*Sinocyclocheilus anshuiensis*, *S. grahami* and *S. rhinocerous*) have two *tyrp1b* copies, which are consistent with their shared lineage-specific whole genome duplication event. Two forms of *tyrp2* were retained in the three golden-line barbel fishes too; however, one *tyrp2* was a pseudogene with a premature stop codon in *S. anshuiensis* and *S. rhinocerous.* On the other hand, the majority of tetrapods have only one copy of *tyr*, *tyrp1*, and *tyrp2*. According to our reconstructed phylogenetic trees for the *tyr*, *tyrp1*, and *tyrp2* genes (Supplementary Figures 1–3), *tyr* genes could be clearly divided into five main groups to represent actinopterygians, amphibians, mammals, reptiles, and birds. Similar to a previous study (Braasch et al., 2007), the *tyr* and *tyrp1* phylogenetic trees (Supplementary Figures 1, 2) clearly revealed that *tyr* and *tyrp1* were doubled in teleosts, consistent with the TWGD event.

### mitf

The *mitf* gene encodes a vital transcription factor that up-regulates *tyr*, *tyrp1*, *tyrp2*, and *pmel* expression for melanin synthesis (Hsiao and Fisher, 2014). Most of tetrapods possess only one *mitf* gene in their genomes (Steingrimsson et al., 2004). In mice, a representative tetrapod model, *mitf* was determined to be related to development of coat color, eye, osteoclasts, and mast cells (Hodgkinson et al., 1993; Widlund and Fisher, 2003; Steingrimsson et al., 2004).

**FIGURE 2:**
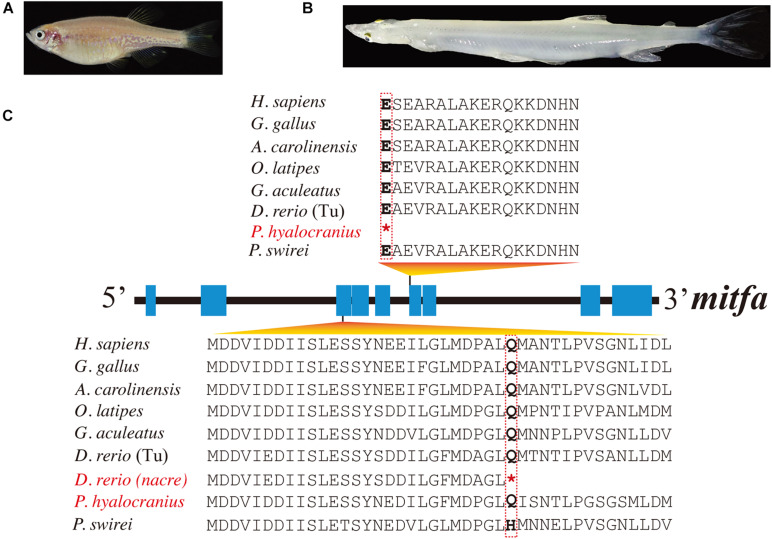
Multiple sequence alignments of *mitfa* of nine representative species. **(A,B)** Photos of transparent *nacre* zebrafish and Chinese clearhead icefish (*P. hyalocranius*). **(C)** Nonsense mutations in the *mitfa* exons of both species. Species with melanin loss are marked in red. *Represents the premature stop codon.

However, duplicated copies of *mitf* genes (named *mitfa* and *mitfb*) are present in the majority of teleost genomes (Steingrimsson et al., 2004). These *mitf* genes have undergone subfunctionalization after genome duplication at least 100 Mya (Altschmied et al., 2002). It was reported that the *mitfa* gene was involved in melanin synthesis, and the *mitfb* gene coexpressed with *mitfa* in the retinal pigment epithelium at an appropriate time to compensate for loss of *mitfa* function in the *nacre* mutant (Lister et al., 2001). A premature stop codon, identified in the *mitfa* exon of *nacre* zebrafish mutants (see more details in Figure 2), caused the loss of melanin pigments in this mutant trunk (Lister et al., 1999; White et al., 2008).

In this study, we identified 135 *mitf* genes in 90 representative vertebrate species (see detailed copy numbers in Supplementary Table 3). Among these examined species, the *mitfa* gene was lost in the cavefish *A. mexicanus*, while it harbored a premature stop codon in *P. hyalocranius* (Figure 2). The phylogenetic tree of *mitfa* and *mitfb* across 90 vertebrate species (Supplementary Figure 4) clearly placed teleosts into one main branch that was split from the common ancestor of teleosts and tetrapods. Subsequently, the teleost branch was divided into *mitfa* and *mitfb* clades, independently.

### pmel

The *pmel* gene (also known as *silver* locus) encodes a type I integral membrane protein that can catalyze eumelanin production (see Figure 1) from indole-5,6-quinone carboxylic acid during melanin synthesis (Chakraborty et al., 1996). Duplication of this gene has been reported in zebrafish (Schonthaler et al., 2005). *pmela* is expressed in melanophores and retinal pigment epithelium, but *pmelb* is expressed exclusively in retinal pigment epithelium (Schonthaler et al., 2005), which is similar to the previously reported subfunctionalization and distributions of the *mitfa* gene and *mitfb* gene in zebrafish (Lister et al., 2001). In mammals, *pmel* transcription is regulated by *mitf* (Du et al., 2003). Several missense mutations in horse *pmel* lead to a phenotype with a characteristic mixture of white and gray hairs (Brunberg et al., 2006). Similarly, a nonsense mutation in the *pmel* gene of Japanese quail caused a completely yellowish plumage phenotype (Ishishita et al., 2018). Furthermore, a 9-bp insertion in the exon 10 of chicken *pmel* led to a *Dominant white* phenotype (Kerje et al., 2004).

**FIGURE 3:**
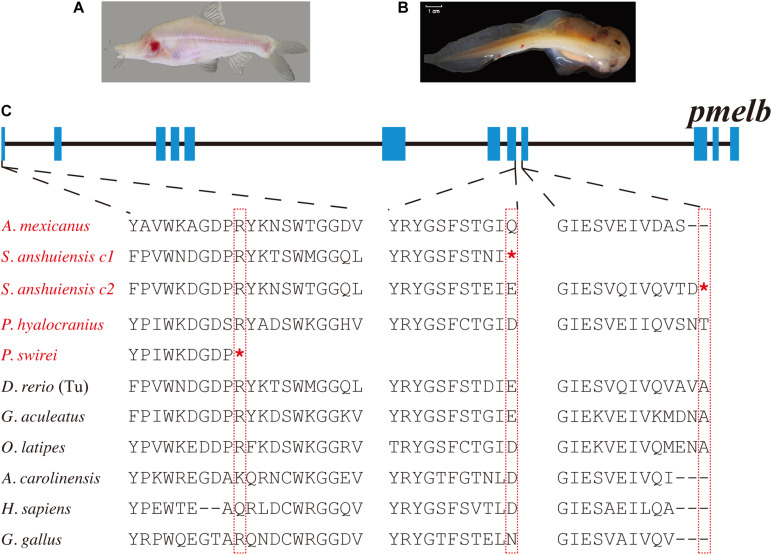
Multiple sequence alignments of *pmelb* in ten representative species. **(A,B)** Photos of a cave-restricted golden-line barbel fish (*S. anshuiensis*) and a deep-sea snailfish (*P. swirei*). This deep-sea snailfish figure was adopted from Wang et al. (2019) with permission. **(C)** Nonsense mutations in the *pmelb* exons of *S. anshuiensis* and *P. swirei.* Species with melanin loss were marked in red. *c1* and *c2* represent copy 1 and copy 2. *Represents the premature stop codon.

In our present study, we found that most teleosts have two *pmel* genes (Supplementary Table 3). Related phylogenetic data confirmed the duplication of *pmel* in teleosts after their divergence from tetrapods (Supplementary Figure 5). In contrast, *pmel* was lost in the majority of birds. Intriguingly, we observed that the *pmel* genes of *P. swirei* and *S. anshuiensis* commonly harbored premature stop codon mutations. More particularly, the two *pmelb* genes were truncated by nonsense mutations in *S. anshuiensis* (see more details in Figure 3).

### slc24a5

*slc24a5*, encoding a transporter protein in the melanosome membrane, is essential for melanin synthesis (Figure 1). It has a pigmentation related function that was firstly identified in teleosts and later in mammals. Loss-of-function mutations in this gene led to reduced melanin concentration in zebrafish (Lamason et al., 2005). However, a non-synonymous mutation in the exon 2 of *slc24a5* in a horse generated bright orange-colored eyes (Mack et al., 2017). A recent study also reported that a non-synonymous mutation in *slc24a5* significantly correlated to skin color in African human populations (Crawford et al., 2017).

**FIGURE 4:**
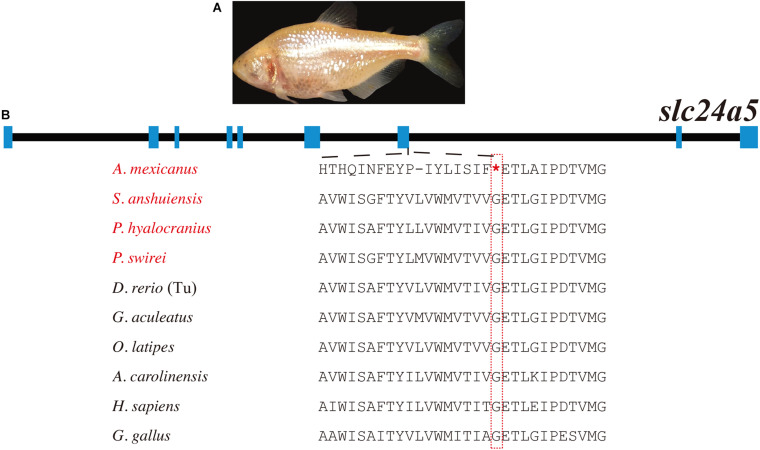
Multiple sequence alignments of *slc24a5* in ten representative species. **(A)** Photo of *A. mexicanus* (cave population), which was downloaded from Wikipedia Commons (NIH Image Gallery, Bethesda, MD, United States). **(B)** A nonsense mutation in the *slc24a5* exon of *A. mexicanus.* Species with melanin loss were marked in red. *Represents the premature stop codon.

In the present study, we identified a total of 94 *slc24a5* genes (Supplementary Tables 3, 4). It has been clearly shown that most of the examined teleosts only have one copy of the *slc24a5* gene, suggesting that its duplicated paralogous gene was lost in the teleost ancestor after the TWGD. The *A. mexicanus* is a unique species that harbors a premature stop codon mutation in its *slc24a5* coding regions (Figure 4 and Table 1). Interestingly, gene copy number and phylogenetic analyses demonstrated the unique presence of three copies of the *slc24a5* gene in Nile tilapia (*Oreochromis niloticus*, Supplementary Figure 6).

## Discussion

### Possible Molecular Clues Regarding the Melanin Loss Phenotype

Animals that are not exposed to light, such as cavefishes, are often colorless or transparent. The cave ecosystem is associated with several traits that are decreased or degenerated over time (i.e., “regressive” traits) (Protas et al., 2006). Notably, the reduced skin pigmentation in cavefishes is an important adaptation that has independently arisen in diverse species, such as *S. anshuiensis* (Yang et al., 2016) and *A. mexicanus* (Gross, 2012).

We identified premature stop variations and gene loss that are potentially leading to melanin loss in fishes with the albinism phenotype, including Mexican tetra (McGaugh et al., 2014), zebrafish (*Danio rerio, nacre/casper* mutants) (Howe et al., 2013; Bian et al., 2020), Chinese clearhead icefish (Liu et al., 2017), deep-sea Mariana snailfish (*Pseudoliparis swirei*), and cave-restricted golden-line barbel fish (*Sinocyclocheilus anshuiensis*; Yang et al., 2016). These premature stop variants and gene loss could provide novel molecular interpretations for melanin loss and albinism in various vertebrates.

Three high-quality genome assemblies of *Sinocyclocheilus* fishes (Cypriniformes: Cyprinidae), including *S. grahami, S. rhinocerous*, and *S. anshuiensis* have been reported before by us (Yang et al., 2016). *S. grahami* is a surface-dwelling species, *S. rhinocerous* is a semi-cave-dwelling species, while *S. anshuiensis* is a cave-restricted counterpart with an albinism phenotype (Figure 3A). These genome data of the three tetraploid fishes were useful for comparative identification of genetic evidence for melanin loss. By comparing these genome assemblies, we found that the *tyrp1a* gene has been lost in the cavefish *S. anshuiensis*. It has been well confirmed that a mis-sense mutation in the *tyrp1a* leads to melanophore death (Krauss et al., 2014). This loss-of-function *tryp1a* could be one potential cue for the melanin loss phenotype in *S. anshuiensis.*

Moreover, both *pmelb* genes in *S. anshuiensis* have a premature stop codon; however, in the other two *Sinocyclocheilus* fish species, *pmelb* genes are commonly normal. In the former case, this may result in functional loss of these genes (to be potential pseudogenes). In fact, many genes for regulating the development of some features may accumulate mutations, ultimately resulting in the loss of functions or even the disappearance of specific traits. The loss of these melanin synthesis genes in *S. anshuiensis* could be important for the white-skin phenotype [15] in this species. A similar nonsense mutation in the *pmelb* coding region is visible in a deep-sea snailfish, *P. swirei*, with a similar melanin loss phenotype (Figure 3). This could be a good example of convergent evolution for mutations in the *pmelb* genes of both white-skinned species that exhibit a melanin pigment loss phenotype.

*A. mexicanus* (cave population) is a perfect diploid model for studying the regressive genetics in cavefishes. A previous study has reported that the albino cave population of *A. mexicanus* harbored deletions in its *oca2* gene (Protas et al., 2006) when compared with its surface counterparts. However, roles of these mutations cannot be critically validated by the gene knockout experiments; in fact, the experimental fish will lose the melanin, when each gene encoding the rate-limiting factors like *mitfa* (Koludrovic and Davidson, 2013), *slc24a5* (Lamason et al., 2005), *oca2* (Protas et al., 2006; Klaassen et al., 2018), *mc1r* (Gross et al., 2009), *tyr* (Kobayashi and Hearing, 2007) for melanin synthesis was knocked out or knocked down. Similarly, the gene gain or loss cannot be correctly identified by only using the QTL (quantitative trait locus) method (Protas et al., 2006) in diverse populations of *A. mexicanus*.

By comparing genes across fish species, we observed that the *A. mexicanus* cave population has lost the *mitfa* gene and harbors a nonsense mutation in *slc24a5*. We therefore speculate that the loss of *mitfa* could be a potential reason for melanin loss in this species and this gene loss may be resulted from several changes. The first step involved the accumulation of mutations in one gene, thereby resulting in missense or nonsense mutations. Since this would have caused a loss of function for this gene, and even the gene eventually disappeared from the genome. Therefore, the loss of *mitfa* gene could have appeared earlier than the nonsense mutation in *slc24a5*. A possible hypothesis could be considered as follows: the loss of *mitfa* led to a defect in the melanin synthesis pathway, resulting in the inactivation and gradual accumulation of neutral mutations in the up- and downstream genes of this pathway.

On the other hand, in addition to the black pigmentation defect, the *A. mexicanus* (cave population) exhibits remarkable yellow or golden skin (Figure 4A), which is similar to the reported *golden* zebrafish mutant with a mutation in *slc24a5*, thereby leading to more lightly pigmented and “golden” fish (Lamason et al., 2005). This similar phenotype provides additional genetic evidence to support the theory that the mutation of *slc24a5* could be possibly involved in this phenotype of *A. mexicanus* (cave population).

Another fish with an albinism phenotype, *P. hyalocranius*, has been reported in our previous paper (Liu et al., 2017). We also found a nonsense mutation in the sixth exon of *mitfa* (Figure 2). It has been well documented that the *mitfa* gene plays a core role in neural crest cell fate specification and melanocyte development (Levy et al., 2006; Wan et al., 2011; Koludrovic and Davidson, 2013). The *nacre/casper* zebrafish strain with a transparent phenotype also harbored a nonsense mutation in its *mitfa* gene (Lister et al., 1999; White et al., 2008). Therefore, the loss-of-function mutation of *mitfa* in *P. hyalocranius* could be the primary molecular mechanism for its melanin loss. Interestingly, the similar mutations in the *mitfa* genes from both *P. hyalocranius* and *nacre/casper* zebrafish were determined, and both fish species present with a similar phenotype of melanin loss in trunk.

## Conclusion

Large numbers of sequenced vertebrate genomes have provided us a great opportunity to perform comparative genomics studies on some interesting genes. We collected 90 representative vertebrate genome assemblies with high quality to analyze detailed copy numbers and gene structures of core genes for melanin synthesis including *tyr, pmel, mitf*, and *slc24a5*. Phylogenetic analysis and genomic alignments were also performed in this study. We identified some novel genetic evidences that loss or nonsense mutations of these core melanin synthesis genes may contribute to melanin loss in these white-skinned fishes. These genetic resources will help to improve the practical breeding of ornamental fishes and create novel transparent models for theoretical researches. Our interesting findings in this study are also instructive for in-depth investigations of human skin coloration.

## Data Availability Statement

The original contributions presented in the study are included in the article/Supplementary Material, further inquiries can be directed to the corresponding authors.

## Ethics Statement

All animal experiments were performed in accordance with the guidelines of the Animal Ethics Committee and were approved by the Institutional Review Board on Bioethics and Biosafety of BGI.

## Author Contributions

QS and WG conceived the project. CB, RL, and ZW performed the data analysis and figure preparation. CB prepared the manuscript. QS and WG revised the manuscript. All authors contributed to the article and approved the submitted version.

## Conflict of Interest

The authors declare that the research was conducted in the absence of any commercial or financial relationships that could be construed as a potential conflict of interest.

## Publisher’s Note

All claims expressed in this article are solely those of the authors and do not necessarily represent those of their affiliated organizations, or those of the publisher, the editors and the reviewers. Any product that may be evaluated in this article, or claim that may be made by its manufacturer, is not guaranteed or endorsed by the publisher.

## Footnotes

1
http://tree.bio.ed.ac.uk/software/figtree
