# Supplementary material for: Phylogenetic Analysis of Core Melanin Synthesis Genes Provides Novel Insights Into the Molecular Basis of Albinism in Fish
Source: Front Genet. 2021 Aug 4;12:707228. doi: 10.3389/fgene.2021.707228 (PMC8371935; doi:10.3389/fgene.2021.707228)
Supplement: Supplementary file 1 [file Data_Sheet_1.docx]

**Table S1.** GenBank ID of the selected 90 vertebrate genomes.

| **Class** | **Species Name** | | **GenBank ID** | | **N50 (bp)** | |  |
| --- | --- | --- | --- | --- | --- | --- | --- |
| **Mammals** | *Ailuropoda melanoleuca* | | GCF_000004335.2 | | 1,281,781 | |  |
|  | *Balaenoptera acutorostrata* | | GCF_000493695.1 | | 12,843,668 | |  |
|  | *Bos taurus* | | GCF_000003055.6 | | 105,708,250 | |  |
|  | *Fukomys damarensis* | | GCF_000743615.1 | | 5,314,287 | |  |
|  | *Homo sapiens* | | GCF_000001405.37 | | 145,138,636 | |  |
|  | *Macaca fascicularis* | | GCF_000364345.1 | | 152,835,861 | |  |
|  | *Mus musculus* | | GCF_000001635.25 | | 130,694,993 | |  |
|  | *Ornithorhynchus anatinus* | | GCF_004115215.1 | | 991,605 | |  |
|  | *Pan paniscus* | | GCF_000258655.2 | | 144,709,823 | |  |
|  | *Physeter catodon* | | GCF_002837175.1 | | 37,183,148 | |  |
|  | *Rattus norvegicus* | | GCF_000001895.5 | | 145,729,302 | |  |
|  | *Tupaia chinensis* | | GCF_000334495.1 | | 3,670,124 | |  |
| **Birds** | *Anas platyrhynchos* | | GCF_000355885.1 | | 1,233,631 | |  |
|  | *Aptenodytes forsteri* | | GCF_000699145.1 | | 5,071,598 | |  |
|  | *Chaetura pelagica* | | GCF_000747805.1 | | 3,841,852 | |  |
|  | *Charadrius vociferus* | | GCF_000708025.1 | | 3,657,050 | |  |
|  | *Corvus brachyrhynchos* | | GCF_000691975.1 | | 6,953,989 | |  |
|  | *Columba livia* | | GCA_000337935.2 | | 3,148,738 | |  |
|  | *Cuculus canorus* | | GCF_000709325.1 | | 2,989,832 | |  |
|  | *Egretta garzetta* | | GCF_000687185.1 | | 3,067,157 | |  |
|  | *Falco peregrinus* | | GCF_000337955.1 | | 3,935,757 | |  |
|  | *Gallus gallus* | | GCA_000002315.4 | | 82,310,166 | |  |
|  | *Geospiza fortis* | | GCF_000277835.1 | | 5,255,844 | |  |
|  | *Haliaeetus leucocephalus* | | GCF_000737465.1 | | 9,145,499 | |  |
|  | *Manacus vitellinus* | | GCF_001715985.1 | | 16,644,750 | |  |
|  | *Meleagris gallopavo* | | GCF_000146605.3 | | 59,006,440 | |  |
|  | *Melopsittacus undulatus* | | GCF_000238935.1 | | 10,614,383 | |  |
|  | *Nestor notabilis* | | GCF_000696875.1 | | 61,475 | |  |
|  | *Nipponia nippon* | | GCF_000708225.1 | | 5,211,696 | |  |
|  | *Opisthocomus hoazin* | | GCA_000692075.1 | | 2,937,227 | |  |
|  | *Picoides_pubescens* | | GCF_000699005.1 | | 2,093,929 | |  |
|  | *Pseudopodoces humilis* | | GCF_000331425.1 | | 16,337,386 | |  |
|  | *Pygoscelis adeliae* | | GCF_000699105.1 | | 5,118,896 | |  |
|  | *Struthio camelus* | | GCF_000698965.1 | | 3,593,425 | |  |
|  | *Taeniopygia guttata* | | GCF_000151805.1 | | 62,374,962 | |  |
|  | *Tinamus guttatus* | | GCF_000705375.1 | | 246,268 | |  |
| **Reptiles** | *Alligator mississippiensis* | | GCF_000281125.3 | | 18,600,682 | |  |
|  | *Alligator sinensis* | | GCF_000455745.1 | | 2,188,296 | |  |
|  | *Anolis carolinensis* | | GCF_000090745.1 | | 150,641,573 | |  |
|  | *Chelonia mydas* | | GCF_000344595.1 | | 3,864,108 | |  |
|  | *Chrysemys picta bellii* | | GCF_000090745.1 | | 7,072,151 | |  |
|  | *Crocodylus porosus* | | GCF_001723895.1 | | 84,437,661 | |  |
|  | *Gavialis gangeticus* | | GCF_001723915.1 | | 96,076,944 | |  |
|  | *Gekko japanicus* | | GCF_001447785.1 | | 707,733 | |  |
|  | *Ophiophagus_hannah* | | GCA_000516915.1 | | 241,519 | |  |
|  | *Pelodiscus sinensis* | | GCF_000230535.1 | | 3,350,749 | |  |
|  | *Python bivittatus* | | GCF_000186305.1 | | 213,970 | |  |
|  | *Thamnophis sirtalis* | | GCF_001077635.1 | | 647,592 | |  |
| **Amphibians** | | *Xenopus laevis* | | GCF_001663975.1 | | 136,570,856 | |
|  | | *Xenopus tropicalis* | | GCF_000004195.3 | | 135,134,832 | |
|  | | *Nanorana parkeri* | | GCF_000935625.1 | | 1,069,101 | |
| **Teleosts** | | *Anabarilius grahami* | | *GCA_003731715.1* | | 4,409,934 | |
|  | | *Astatotilapia burtoni* | | *GCF_000239415.1* | | 1,194,190 | |
|  | | *Astyanax mexicanus* | | *GCF_000372685.1* | | 1,775,308 | |
|  | | *Boleophthalmus pectinirostris* | | *GCF_000788275.1* | | 2,375,582 | |
|  | | *Coilia nasus* | | *GCA_007927635.1* | | 34,854,322 | |
|  | | *Ctenopharyngodon idellus* | | [*PRJEB5920*](http://www.ncbi.nlm.nih.gov/bioproject/?term=PRJEB5920) | | 6,428,943 | |
|  | | *Cynoglossus semilaevis* | | *GCF_000523025.1* | | 20,010,643 | |
|  | | *Cyprinus carpio* | | *GCF_000951625.1* | | 66,838 | |
|  | | *Danio rerio* | | *GCF_000002035.4* | | 53,345,113 | |
|  | | *Dicentrarchus labrax* | | *GCA_000689215.1* | | 26,439,989 | |
|  | | *Esox lucius* | | *GCA_000721915.3* | | 32,939,842 | |
|  | | *Fundulus heteroclitus* | | *GCF_000826765.1* | | 1,252,252 | |
|  | | *Gasterosteus aculeatus* | | *GCA_000180675.1* | | 83,204 | |
|  | | *Gadus morhua* | | *GCF_902167405.1* | | 393,166 | |
|  | | *Hippocampus comes* | | *GCF_001891065.1* | | 1,971,390 | |
|  | | *Ictalurus punctatus* | | [*GCF_001660625.1*](https://www.ncbi.nlm.nih.gov/assembly/763271) | | 27,425,808 | |
|  | | *Larimichthys crocea* | | *GCF_000972845.1* | | 1,034,540 | |
|  | | *Lates calcarifer* | | *GCF_001640805.1* | | 1,191,366 | |
|  | | *Lepisosteus oculatus* | | *GCF_000242695.1* | | 50,348,508 | |
|  | | *Monopterus albus* | | *GCF_001952655.1* | | 2,106,322 | |
|  | | *Neolamprologus brichardi* | | *GCF_000239395.1* | | 4,430,025 | |
|  | | *Oncorhynchus mykiss* | | *GCF_002163495.1* | | 71,056,191 | |
|  | | *Oreochromis niloticus* | | *GCF_001858045.1* | | 37,007,722 | |
|  | | *Oryzias latipes* | | *GCF_000313675.1* | | 29,600,056 | |
|  | | *Periophthalmus magnuspinnatus* | | *GCA_000787105.1* | | 296,161 | |
|  | | *Poecilia formosa* | | *GCF_000485575.1* | | 1,574,226 | |
|  | | *Protosalanx hyalocranius* | | *PRJNA328051* | | 432,316 | |
|  | | *Pseudoliparis swirei* | | *PRJNA472845* | | 418,461 | |
|  | | *Pygocentrus nattereri* | | *GCF_001682695.1* | | 1,440,044 | |
|  | | *Salmo salar* | | *GCF_000233375.4* | | 80,503,876 | |
|  | | *Sinocyclocheilus anshuiensis* | | *GCF_001515605.1* | | 1,284,143 | |
|  | | *Sinocyclocheilus grahami* | | *GCF_001515645* | | 1,156,368 | |
|  | | *Sinocyclocheilus rhinocerous* | | *GCF_001515625* | | 945,738 | |
|  | | *Takifugu rubripes* | | *GCF_000180615.1* | | 11,516,971 | |
|  | | *Tetraodon nigroviridis* | | *GCA_000180735.1* | | 734,039 | |
|  | | *Xiphophorus maculatus* | | *GCF_000241075.1* | | 1,303,070 | |
| **Chondrichthyes** | | *Callorhynchus milii* | | GCF_000165045.1 | | 4,521,921 | |

**Table S2.** Queries used for extraction of genes from the melanin synthesis pathway.

| **Gene** | ***O. latipes*** | ***H. sapiens*** | ***G. gallus*** | ***X. laevis*** | ***A. carolinensis*** |
| --- | --- | --- | --- | --- | --- |
| ***tyra/tyr*** | ENSORLP00000041517 | ENSP00000263321 | ENSP00000263321 | ENSACAP00000014741 | ENSACAP00000014741 |
| ***tyrb*** | ENSORLP00000005706 |  |  |  |  |
| ***tyrp1a/tyrp1*** | ENSORLP00000005420 | ENSP00000373570 | ENSP00000373570 | ENSACAP00000011189 | ENSACAP00000011189 |
| ***tyrp1b*** | ENSORLP00000033423 |  |  |  |  |
| ***tyrp2*** | ENSORLP00000022234 | ENSP00000392762 | ENSGALP00000035733 | ENSXETP00000023918 | ENSACAP00000002600 |
| ***pmela/pmel*** | ENSORLP00000018803 | ENSP00000402758 | ENSGALP00000051268 | ENSXETP00000020810 | ENSACAP00000005360 |
| ***pmelb*** | ENSORLP00000020625 |  |  |  |  |
| ***mitfa/mitf*** | ENSORLP00000037247 | ENSP00000391803 | ENSGALP00000070157 | ENSXETP00000000313 | ENSACAP00000013404 |
| ***mitfb*** | ENSORLP00000033348 |  |  |  |  |
| ***slc24a5*** | ENSORLP00000045188 | ENSP00000341550 | ENSGALP00000038183 | ENSXETP00000039854 | ENSACAP00000004671 |

**Table S3.** Copy numbers of genes for melanin synthesis in 38 examined Actinopterygii species.

| **species** | ***tyra*** | ***tyrb*** | ***tyrp1a*** | ***tyrp1b*** | ***tyrp2*** | ***pmela*** | ***pmelb*** | ***mitfa*** | ***mitfb*** | ***slc24a5*** |
| --- | --- | --- | --- | --- | --- | --- | --- | --- | --- | --- |
| *Anabarilius_grahami* | 1 | 0 | 1 | 1 | 1 | 1 | 1 | 1 | 1 | 1 |
| *Astatotilapia_burtoni* | 1 | 1 | 1 | 1 | 1 | 1 | 1 | 1 | 1 | 1 |
| *Astyanax_mexicanus* | 1 | 1 | 1 | 1 | 1 | 1 | 1 | 0 | 0 | 0 (1) |
| *Boleophthalmus_pectinirostris* | 1 | 1 | 1 | 1 | 1 | 1 | 1 | 1 | 1 | 1 |
| *Coilia_nasus* | 1 | 0 | 1 | 1 | 1 | 1 | 1 | 1 | 1 | 1 |
| *Ctenopharyngodon_idellus* | 1 | 0 | 1 | 1 | 1 | 1 | 2 | 1 | 1 | 1 |
| *Cynoglossus_semilaevis* | 1 | 1 | 1 | 1 | 1 | 1 | 1 | 1 | 1 | 1 |
| *Cyprinus_carpio* | 2 | 0 | 1 | 1 | 1 | 0 (1) | 1 | 1 | 2 | 1 |
| *Danio_rerio* | 1 | 0 | 1 | 1 | 1 | 1 | 1 | 1 | 1 | 1 |
| *Dicentrarchus_labrax* | 1 | 1 | 1 | 1 | 1 | 1 | 1 | 1 | 1 | 1 |
| *Esox_lucius* | 1 | 1 | 1 | 1 | 1 | 1 | 1 | 1 | 1 | 1 |
| *Fundulus_heteroclitus* | 1 | 1 | 1 | 1 | 1 | 1 | 1 | 1 | 1 | 1 |
| *Gasterosteus_aculeatus* | 1 | 1 | 1 | 1 | 1 | 1 | 1 | 1 | 1 | 1 |
| *Guadus_morhua* | 0 | 1 | 1 | 1 | 0 | 1 | 1 | 1 | 1 | 1 |
| *Hippocampus_comes* | 1 | 1 | 1 | 1 | 1 | 1 | 1 | 1 | 1 | 1 |
| *Ictalurus_punctatus* | 1 | 1 | 1 | 1 | 1 | 1 | 1 | 1 | 0 | 1 |
| *Larimichthys_crocea* | 1 | 0 | 1 | 1 | 1 | 2 | 1 | 1 | 1 | 1 |
| *Lates_calcarifer* | 1 | 1 | 1 | 1 | 1 | 2 | 1 | 2 | 1 | 1 |
| *Monopterus_albus* | 1 | 1 | 1 | 1 | 1 | 1 | 1 | 1 | 2 | 1 |
| *Neolamprologus_brichardi* | 1 | 1 | 1 | 1 | 1 | 1 | 1 | 1 | 1 | 1 |
| *Oncorhynchus_mykiss* | 1 | 1 | 2 | 1 | 2 | 1 | 2 | 1 | 3 | 1 |
| *Oreochromis_niloticus* | 1 | 1 | 1 | 1 | 1 | 1 | 1 | 1 | 1 | 3 |
| *Oryzias_latipes* | 1 | 1 | 1 | 1 | 1 | 1 | 1 | 1 | 1 | 1 |
| *Periophthalmus_magnuspinnatus* | 1 | 1 | 1 | 1 | 1 | 1 | 1 | 1 | 1 | 1 |
| *Poecilia_formosa* | 1 | 1 | 1 | 1 | 1 | 1 | 1 | 1 | 1 | 1 |
| *Protosalanx_hyalocranius* | 1 | 1 | 1 | 1 | 1 | 1 | 1 | 0 (1) | 1 | 1 |
| *Pseudoliparis_swirei* | 0 | 2 | 1 | 1 | 1 | 1 | 0 (1) | 1 | 1 | 1 |
| *Pundamilia_nyererei* | 1 | 1 | 1 | 1 | 1 | 1 | 1 | 1 | 1 | 1 |
| *Pygocentrus_nattereri* | 1 | 1 | 1 | 1 | 1 | 1 | 1 | 1 | 1 | 1 |
| *Salmo_salar* | 1 | 2 | 2 | 0 | 2 | 2 | 2 | 1 | 2 | 1 |
| *Sinocyclocheilus_anshuiensis* | 2 | 0 | 0 | 2 | 1 (1) | 2 | 0 (2) | 1 | 2 | 1 |
| *Sinocyclocheilus_grahami* | 2 | 0 | 1 | 2 | 2 | 2 | 1 | 1 | 1 | 2 |
| *Sinocyclocheilus_rhinocerous* | 1 | 0 | 1 | 2 | 1 (1) | 1 | 1 (1) | 1 | 2 | 1 |
| *Takifugu_rubripes* | 1 | 1 | 1 | 0 | 1 | 1 | 1 | 1 | 1 | 1 |
| *Tetraodon_nigroviridis* | 1 | 1 | 1 | 0 | 1 | 1 | 1 | 1 | 1 | 1 |
| *Xiphophorus_maculatus* | 1 | 1 | 1 | 1 | 1 | 1 | 1 | 1 | 1 | 1 |
| **Total** | **37** | **29** | **37** | **36** | **38 (2)** | **40 (1)** | **37 (4)** | **39 (1)** | **41** | **38 (1)** |

Those species with melanin loss were marked in red.

The copy numbers of pseudogenes were provided in brackets.

**Table S4.** Copy numbers of genes for melanin synthesis in 53 examined tetrapod species.

| **Class** | **species** | ***tyr*** | ***tyrp1*** | ***tyrp2*** | ***pmel*** | ***mitf*** | ***slc24a5*** |
| --- | --- | --- | --- | --- | --- | --- | --- |
| **Mammals** | *Ailuropoda_melanoleuca* | 0 | 1 | 1 | 1 | 1 | 1 |
|  | *Balaenoptera_acutorostrata* | 1 | 1 | 1 | 1 | 1 | 1 |
|  | *Bos_taurus* | 1 | 1 | 1 | 1 | 1 | 1 |
|  | *Castor_canadensis* | 0 | 1 | 1 | 1 | 1 | 1 |
|  | *Fukomys_damarensis* | 1 | 0 | 1 | 1 | 1 | 1 |
|  | *Homo_sapiens* | 1 | 1 | 1 | 1 | 1 | 1 |
|  | *Macaca_fascicularis* | 1 | 1 | 1 | 1 | 1 | 1 |
|  | *Mus_musculus* | 1 | 1 | 1 | 1 | 1 | 1 |
|  | *Ornithorhynchus_anatinus* | 1 | 1 | 1 | 1 | 1 | 1 |
|  | *Pan_paniscus* | 1 | 1 | 1 | 1 | 1 | 1 |
|  | *Physeter_catodon* | 1 | 1 | 0 (1) | 1 | 1 | 1 |
|  | *Rattus_norvegicus* | 1 | 1 | 1 | 1 | 1 | 1 |
|  | *Tupaia_chinensis* | 1 | 1 | 1 | 1 | 1 | 1 |
|  | **Total** | **11** | **12** | **12 (1)** | **13** | **13** | **13** |
| **Birds** | *Anas_platyrhynchos* | 1 | 1 | 1 | 0 | 1 | 1 |
|  | *Aptenodytes_forsteri* | 1 | 1 | 1 | 0 | 1 | 1 |
|  | *Calypte_anna* | 1 | 1 | 1 | 0 | 1 | 1 |
|  | *Chaetura_pelagica* | 1 | 1 | 1 | 0 | 1 | 1 |
|  | *Charadrius_vociferus* | 1 | 1 | 0 (1) | 0 | 1 | 1 |
|  | *Columba_livia* | 1 | 1 | 1 | 1 | 1 | 1 |
|  | *Corvus_brachyrhynchos* | 1 | 1 | 1 | 0 | 1 | 1 |
|  | *Cuculus_canorus* | 1 | 1 | 1 | 0 | 1 | 1 |
|  | *Egretta_garzetta* | 1 | 1 | 1 | 0 | 1 | 1 |
|  | *Falco_peregrinus* | 1 | 1 | 1 | 0 | 1 | 1 |
|  | *Gallus_gallus* | 1 | 1 | 1 | 1 | 1 | 1 |
|  | *Geospiza_fortis* | 1 | 1 | 1 | 0 | 1 | 1 |
|  | *Haliaeetus_leucocephalus* | 1 | 1 | 1 | 1 | 1 | 1 |
|  | *Manacus_vitellinus* | 1 | 1 | 1 | 0 | 1 | 1 |
|  | *Meleagris_gallopavo* | 1 | 1 | 1 | 1 | 1 | 1 |
|  | *Melopsittacus_undulatus* | 1 | 1 | 1 | 0 | 1 | 1 |
|  | *Nestor_notabilis* | 1 | 1 | 1 | 0 | 1 | 1 |
|  | *Nipponia_nippon* | 1 | 1 | 0 (1) | 0 | 1 | 1 |
|  | *Opisthocomus_hoazin* | 1 | 1 | 1 | 0 | 1 | 1 |
|  | *Picoides_pubescens* | 1 | 1 | 1 | 0 | 1 | 1 |
|  | *Pseudopodoces_humilis* | 1 | 1 | 1 | 1 | 1 | 1 |
|  | *Pygoscelis_adeliae* | 1 | 1 | 1 | 0 | 1 | 1 |
|  | *Struthio_camelus* | 1 | 1 | 1 | 0 | 1 | 1 |
|  | *Taeniopygia_guttata* | 1 | 1 | 1 | 0 | 1 | 1 |
|  | *Tinamus_guttatus* | 1 | 1 | 1 | 0 | 1 | 1 |
|  | **Total** | **25** | **25** | **23(2)** | **5** | **25** | **25** |
| **Reptiles** | *Alligator_mississippiensis* | 1 | 1 | 1 | 1 | 1 | 1 |
|  | *Alligator_sinensis* | 1 | 1 | 1 | 1 | 1 | 1 |
|  | *Anolis_carolinensis* | 1 | 1 | 1 | 1 | 1 | 1 |
|  | *Chelonia_mydas* | 1 | 1 | 1 | 0 | 1 | 1 |
|  | *Chrysemys_picta* | 1 | 1 | 1 | 1 | 1 | 1 |
|  | *Crocodylus_porosus* | 1 | 1 | 1 | 0 | 1 | 1 |
|  | *Gavialis_gangeticus* | 1 | 1 | 1 | 0 | 1 | 1 |
|  | *Gekko_japonicus* | 1 | 1 | 1 | 1 | 1 | 1 |
|  | *Ophiophagus_hannah* | 0 | 1 | 1 | 1 | 1 | 1 |
|  | *Pelodiscus_sinensis* | 1 | 1 | 1 | 0 | 1 | 1 |
|  | *Python_bivittatus* | 0 | 1 | 1 | 1 | 1 | 1 |
|  | *Thamnophis_sirtalis* | 0 | 1 | 1 | 1 | 1 | 1 |
|  | **Total** | **9** | **12** | **12** | **8** | **12** | **12** |
| **Amphibians** | *Nanorana_parkeri* | 1 | 2 | 1 | 1 | 1 | 1 |
|  | *Xenopus_laevis* | 1 | 2 | 2 | 2 | 2 | 2 |
|  | *Xenopus_tropicalis* | 1 | 1 | 1 | 1 | 1 | 1 |
|  | **Total** | **3** | **5** | **4** | **4** | **4** | **4** |

**Table S5.** The detailed locations in scaffolds/chromosomes for genes from the melanin synthesis pathway in various fishes.

(*See the separated Excel file*)

**Table S6**. The detailed locations in scaffolds/chromosomes for genes from melanin synthesis pathway in various tetrapod species.

(*See the separated Excel file*)

**Supplementary File 1**. The perl script named Solar.pl used for tBLASTn alignment filtering.

**Supplementary File 2**. Coding sequences of all reported genes in this study.

(*See the separated compressed files*)

**Supplementary File 3**. Protein sequence alignments of reported genes in this study.

(*See the separated compressed files*)

**Supplementary File 4**. Protein sequence alignments of *mitfa, pmelb,* and *slc24a5* genes in Figures 2, 3 and 4.

(*See the separated compressed files*)

**
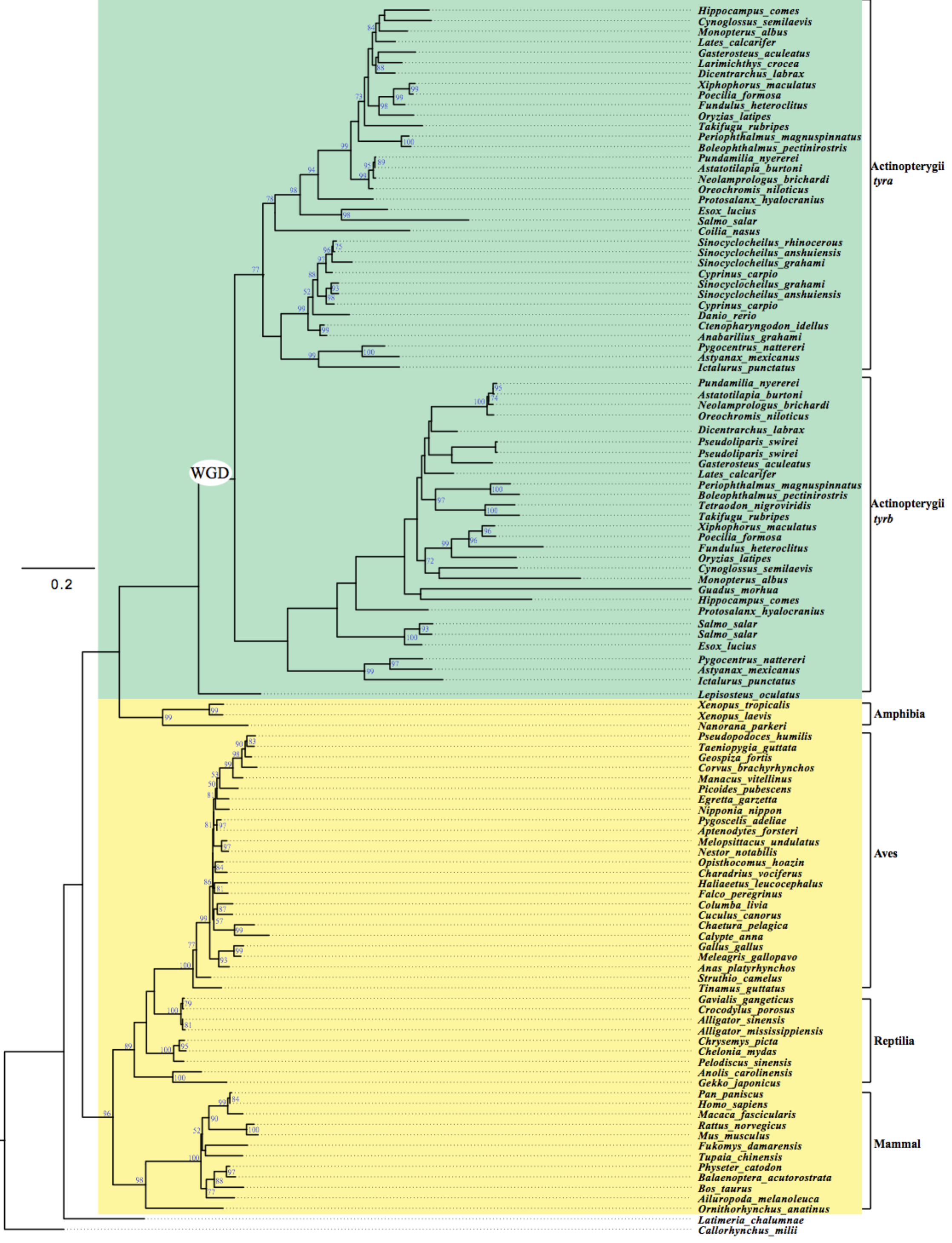
**

**Figure S1.** A phylogenetic tree of *tyr* in various vertebrates. The scale bar denotes substitutions per site. Bootstrap values above 50% were provided at the branches.


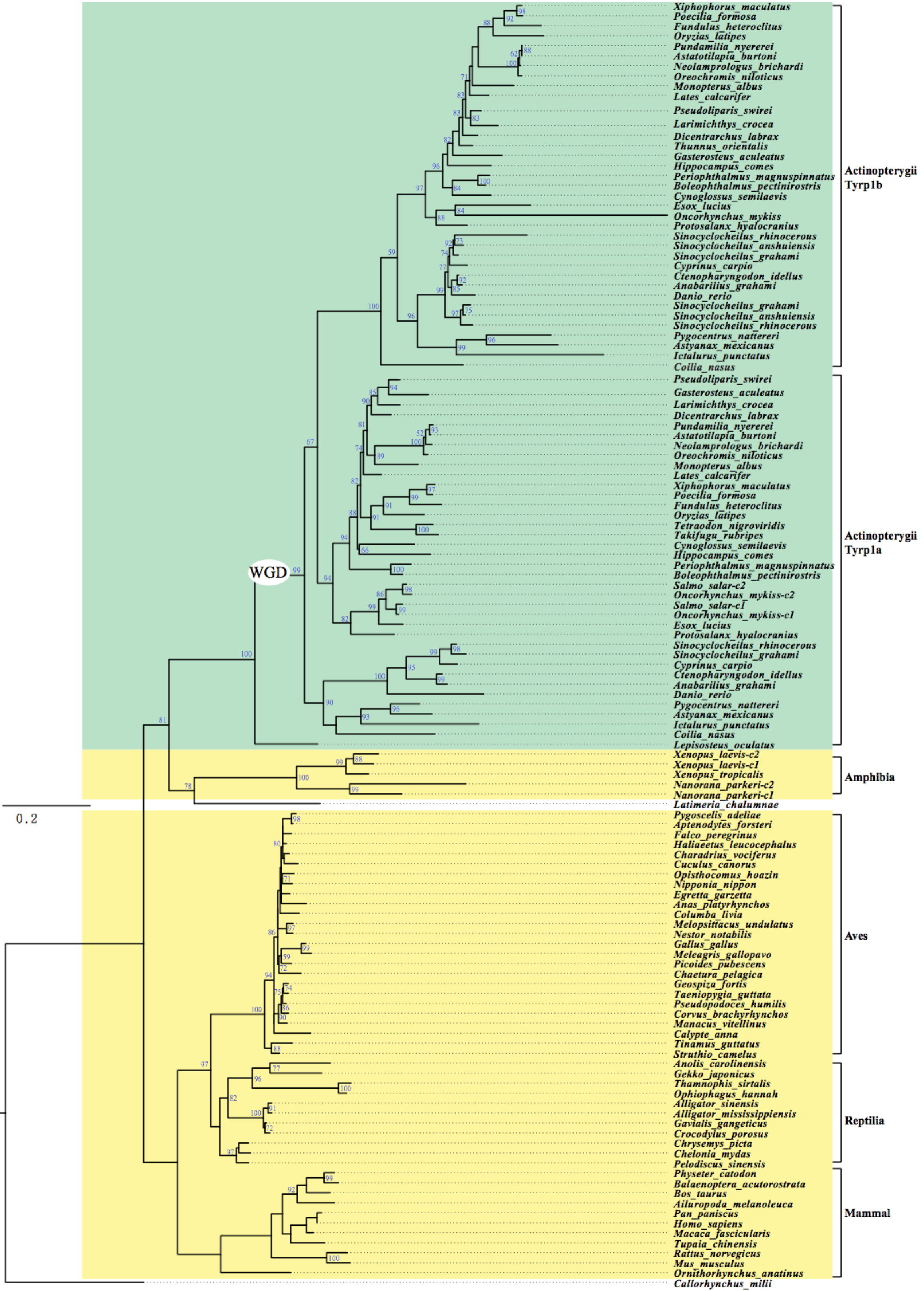


**Figure S2.** A phylogenetic tree of *tyrp1* in various vertebrates. The scale bar denotes substitutions per site. Bootstrap values above 50% were provided at the branches.


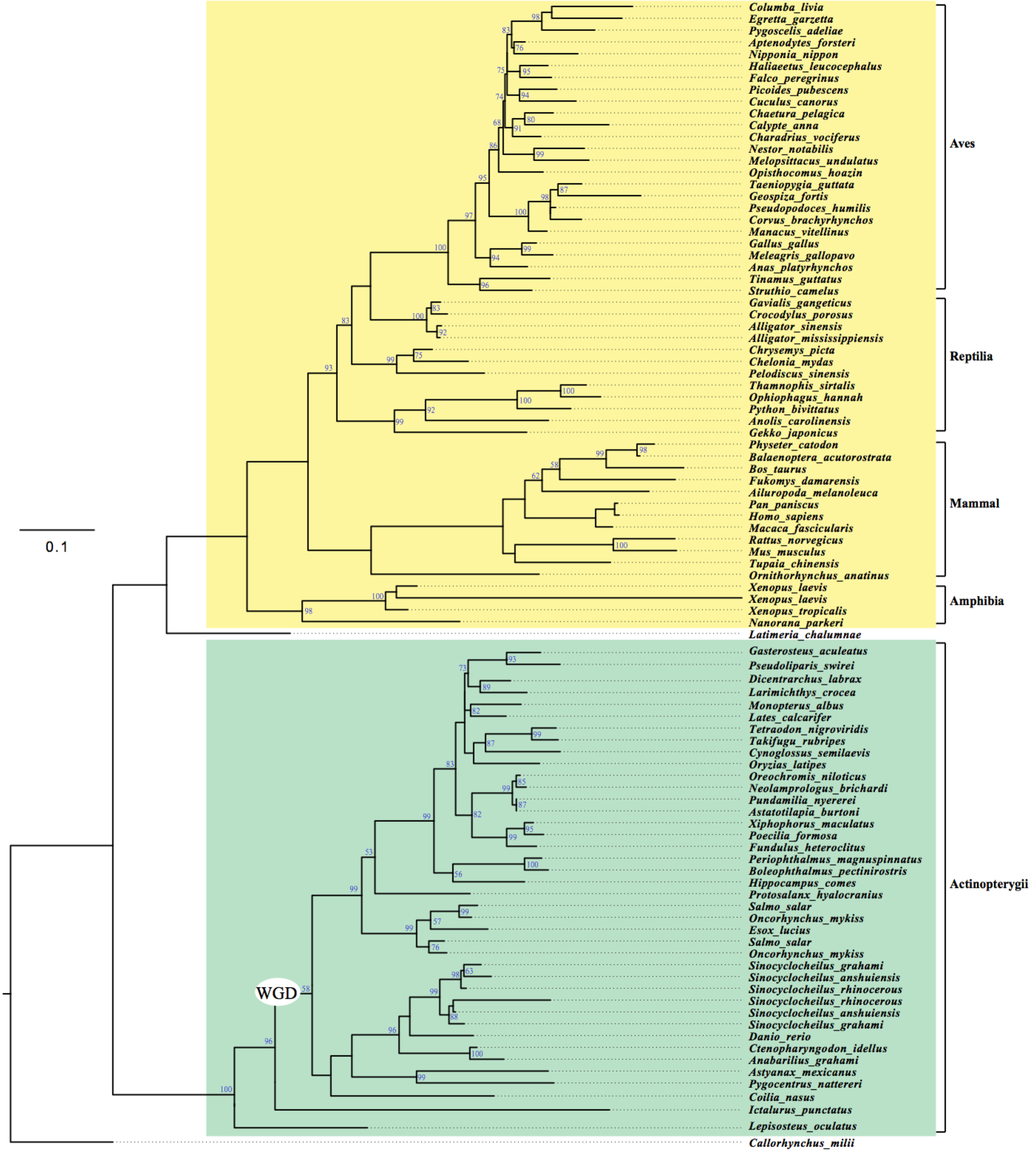


**Figure S3.** A phylogenetic tree of *tyrp2* in various vertebrates. The scale bar denotes substitutions per site. Bootstrap values above 50% were provided at the branches.


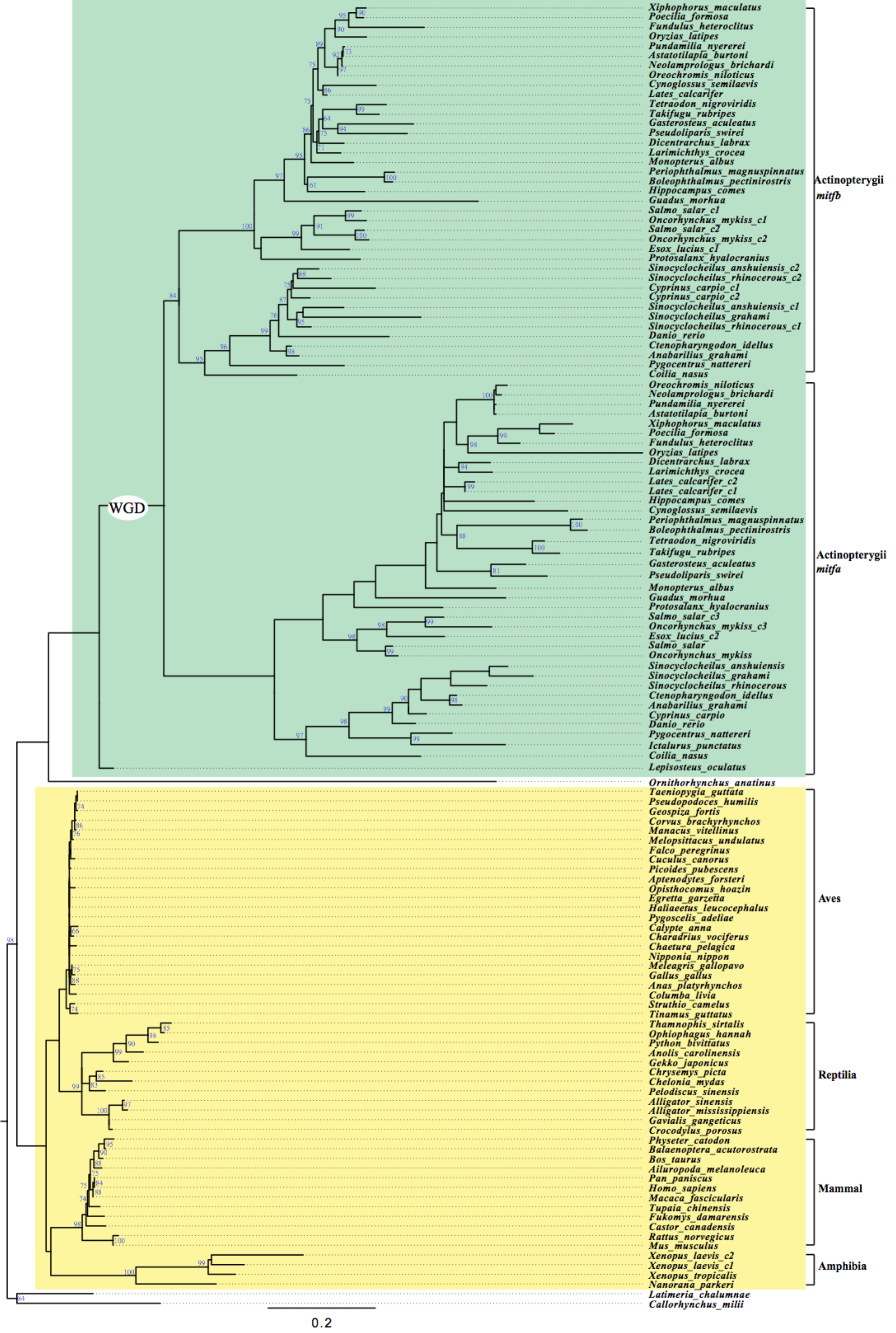


**Figure S4.** A phylogenetic tree of *mitf* in various vertebrates. The scale bar denotes substitutions per site. Bootstrap values above 50% were provided at the branches.


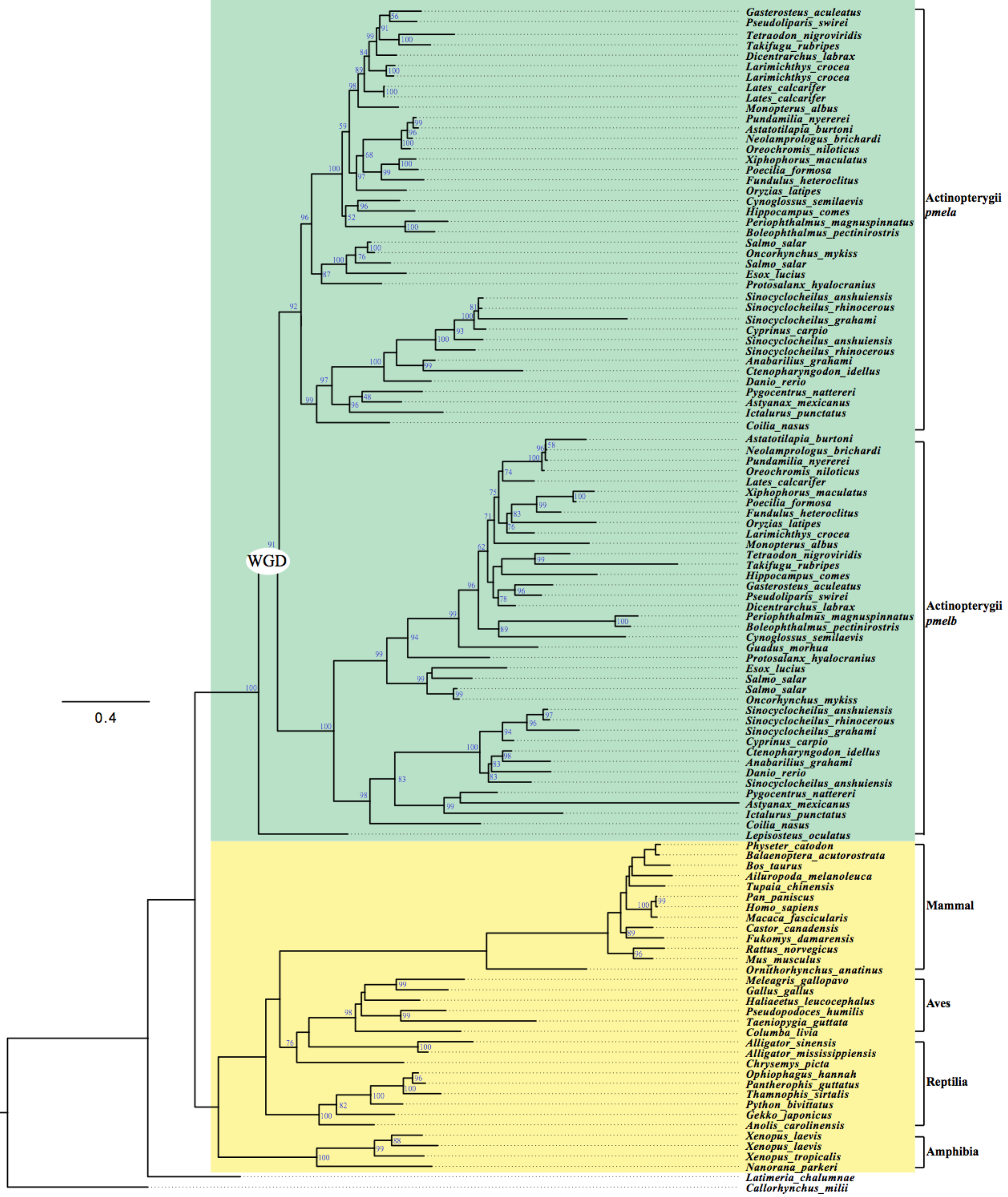


**Figure S5.** A phylogenetic tree of *pmel* in various vertebrates. The scale bar denotes substitutions per site. Bootstrap values above 50% were provided at the branches.


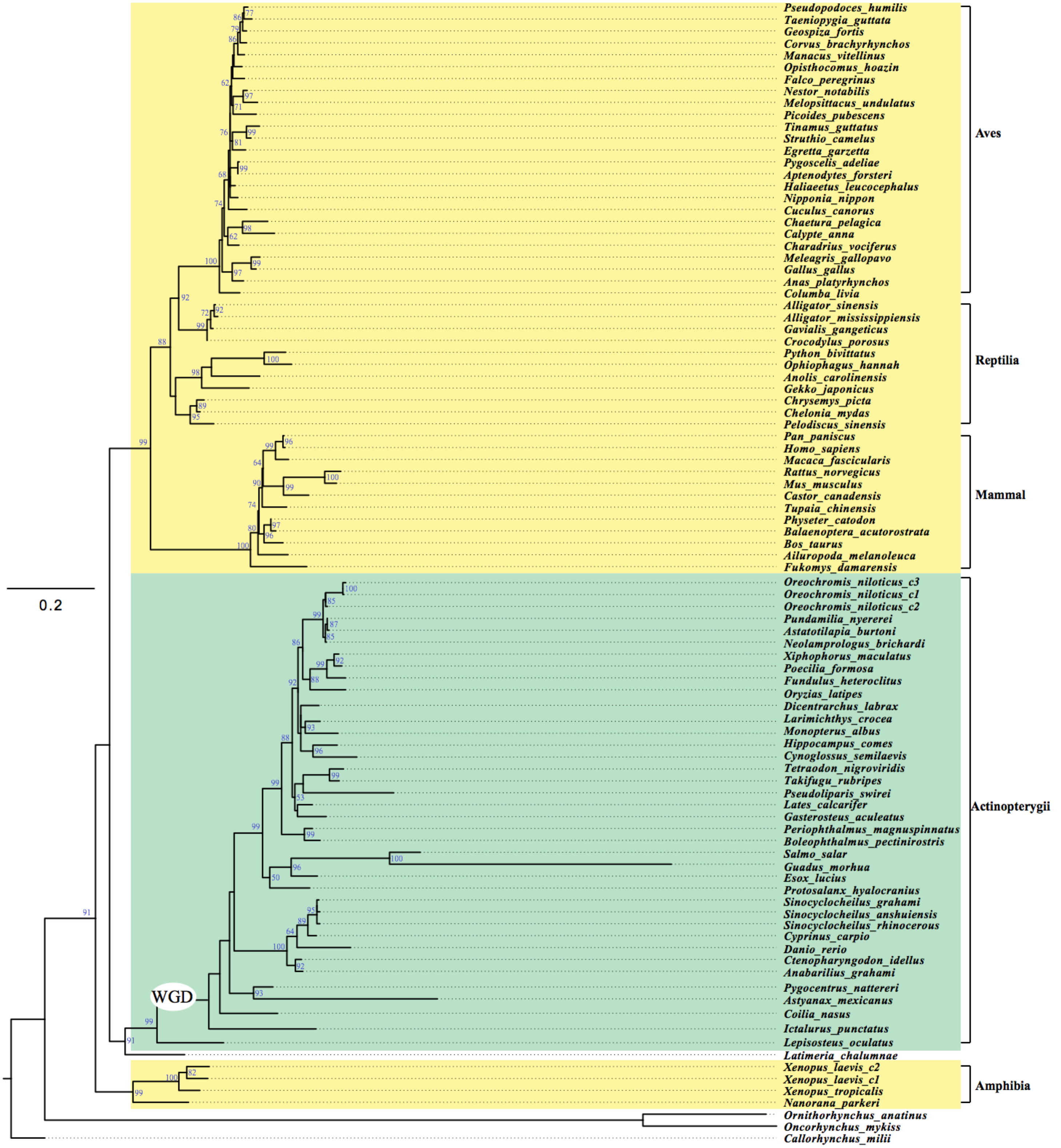


**Figure S6.** A phylogenetic tree of *slc24a5* in various vertebrates. The scale bar denotes substitutions per site. Bootstrap values above 50% were provided at the branches.
